# The first case report of multiple thoracic vertebrae fractures caused by a low-voltage electric shock

**DOI:** 10.1186/s40001-022-00681-4

**Published:** 2022-04-04

**Authors:** Jan Žatecký, Matúš Peteja, Wladyslaw Bartosz Gawel, Milan Lerch

**Affiliations:** 1 The Institute of Paramedical Health Studies, Faculty of Public Policies, Silesian University in Opava, Bezručovo náměstí 14, Opava, Czech Republic; 2 Department of Surgery, Silesian Hospital in Opava, Olomoucká 470/86, 746 01 Opava, Czech Republic

**Keywords:** Multiple vertebrae fractures, Electric shock, Electric injury, Trauma, Support corset

## Abstract

**Background:**

This paper describes a unique case—the first case of multiple fractures of the thoracic vertebrae caused by a low-voltage electric shock.

**Case presentation:**

A 22-year-old male patient was diagnosed with compression fractures of Th2–Th6 caused by a muscle spasm resulting from an electric shock. The patient was treated conservatively using a cervico-thoracic support corset. After rehabilitation, the patient has regained his physiological movement of the spine without any back pain.

**Conclusions:**

Albeit vertebral fractures caused by electric shock injury are extremely rare, clinicians should always keep in mind this diagnosis, especially when clinical symptoms such as pain and limitation of movement are present.

## Background

Vertebral fractures (VF) are among the most common injuries, mainly affecting the thoracic and lumbar regions (T12 or L1) [1, 2]. The prevalence is growing with age, achieving the maximum in the population over 70 years (20%) [3]. While high energy injury is the most common mechanism, it must be noted that other mechanisms may also result in a vertebral fracture (VF) [4]. One of the less obvious mechanisms involves the tetanic muscle spasm caused by an electric shock [5]. Although the vast majority of fractures in this mechanism are not caused by the electric injury itself but rather by a subsequent fall, one must be vigilant when examining the patient after low-voltage (LV) shock. Herein, we describe the first case of multiple VF following a muscle spasm resulting from LV trauma.

## Case presentation

A 22-year-old male patient, with no prior medical history, was admitted to the trauma ward of the Department of Surgery after suffering an electric shock by the guitar combo amplifier. The patient suffered a full-body spasm for a few seconds, after which he unplugged the amplifier and asked his mother to call an ambulance. The patient remained conscious and did not have a fall after the electric shock.

**Fig. 1:**
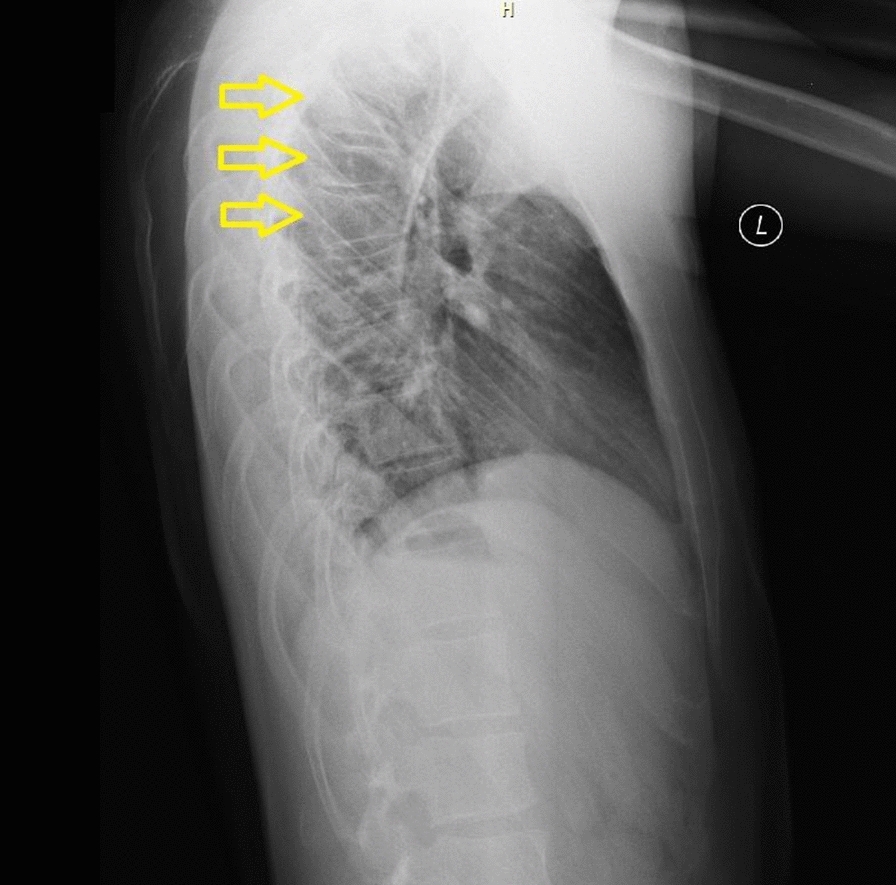
X-ray of thoracic vertebrae revealing Th3–5 compression fractures (yellow arrows)

**Fig. 2:**
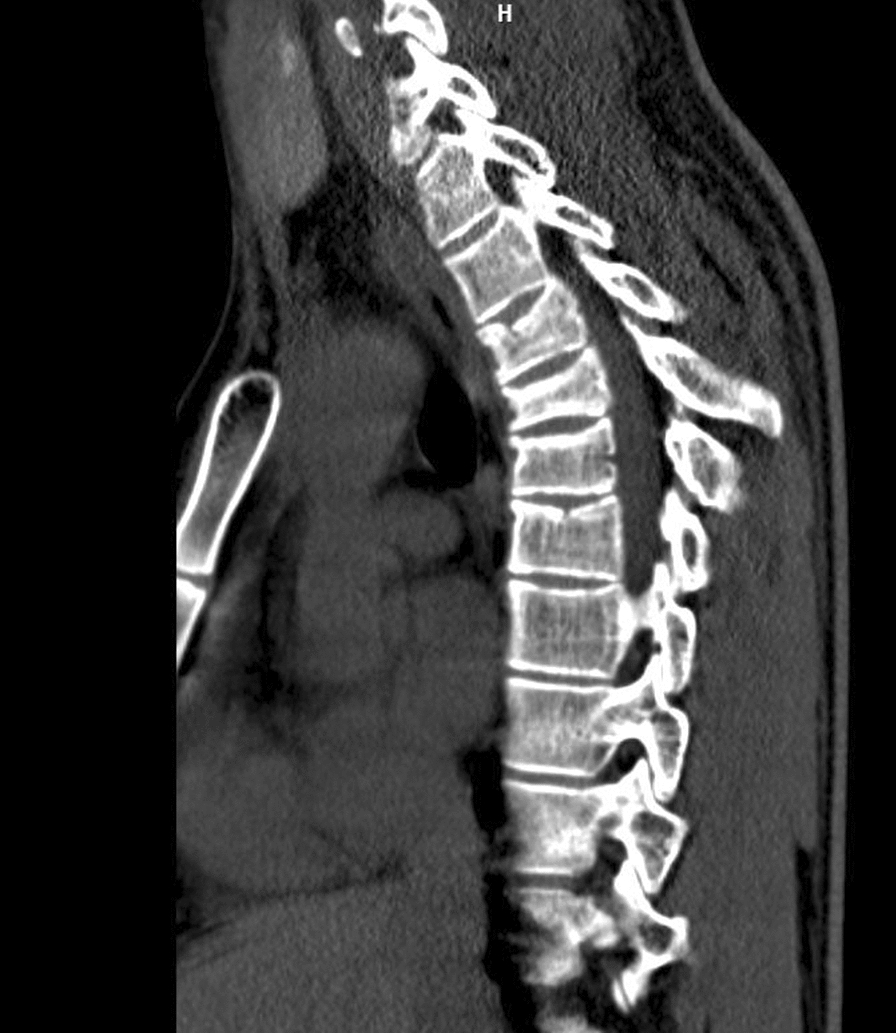
CT scan showing multiple compression fractures of thoracic vertebrae

Clinical examination revealed a second-degree burn on the fifth finger of the right hand (3 × 5 mm) and a minor burn on the thumb of the left hand. The patient reported pain in the interscapular region but the clinical examination did not reveal any other pathologies. X-ray, however, revealed compression fractures of Th3–Th5 (Fig. 1). Following these findings, a CT scan was performed confirming the presence of compression fractures of the aforementioned vertebrae; the spinal cavity was intact (Fig. 2). Transthoracic echocardiography revealed no pathology.

**Fig. 3:**
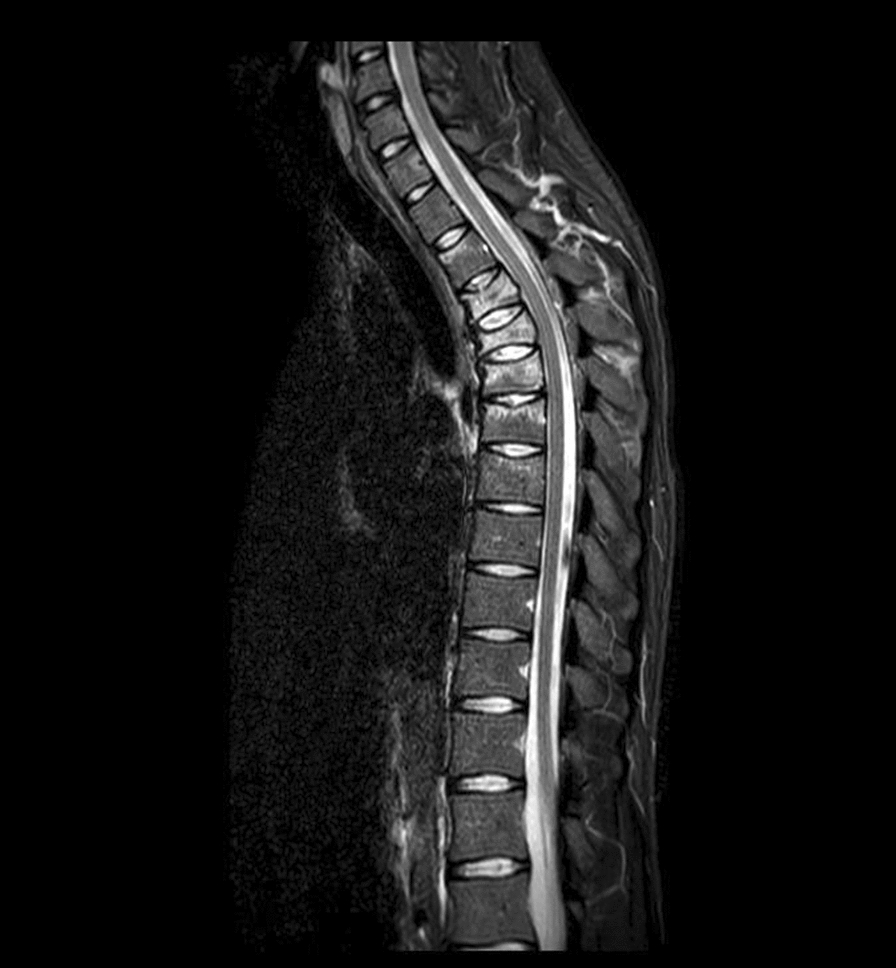
MRI scan confirming multiple compression fractures of thoracic vertebrae

**Fig. 4:**
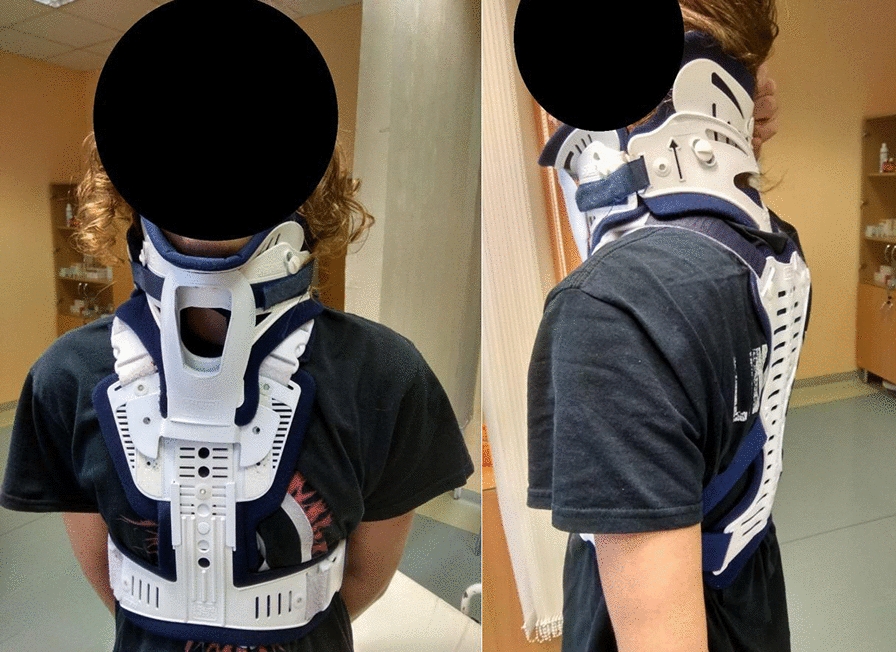
Patient in a cervico-thoracic support corset (Miami JTO)

Following the radiological findings, neurological and neurosurgical consultations were performed. The neurosurgeon recommended MRI to rule out injuries to the spinal cord and ligaments. MRI revealed a compression fracture of the 3rd (T3: A3; according to AO thoracolumbar classification system), 4th (T4: A3), 5th (T5: A1) and 6th (T6: A1) thoracic vertebrae. In addition, MRI detected also a small compression of the 2nd thoracic vertebra (T2: A1) and lesions of interspinous ligaments Th2–Th5 (Fig. 3). Based on the MRI results, conservative treatment using a cervico-thoracic support corset (Miami JTO) (Fig. 4.) was prescribed. The posterior ligamentous complex (PLC) was, according to MRI scans, intact. Neurological examination did not reveal any neurological deficit (N0). The analgesic therapy along with rehabilitation resulted in verticalization of the patient on day + 11 after a standing X-ray proving no deterioration of fractures nor progression of kyphosis. The patient was discharged on day + 12.

At the follow-up visit 1 month after the injury, the patient complained of moderate pain in the back; an X-ray was performed but no kyphotisation was present. MRI scan at 2.5 months was confirmed the reparative changes of the Th2–Th6 fractures without any sign of a spinal injury; therefore, a decision was made to withdraw a corset and commence rehabilitation, which lasted for 2.5 months and resulted in the full restoration of the movement and eased the pain.

## Discussion

The VF resulting from electric shock are mainly connected with high voltage (HV) electricity and concomitant falling from the height [6]. The muscle spasm following the spontaneous LV electric shock, as opposed to electric shock therapy-induced fracture [7], is an extremely rare mechanism of injury.

The literature review provided only a handful of such cases, none of which included multiple VF.

Brink and Leeuwen [8] reported a similar case of lumbar burst fracture due to a muscle spasm following an LV shock, while Putti et al. [9] described a C5 fracture. Vincenti et al. [10] reported 2 more cases of VF associated with an electric shock. An overview of related papers is presented in Table 1.

**Table 1:** List of publications describing vertebral fractures resulting from low-voltage electric shock

Authors	Year of publication	Injury	Mechanism of injury	Assoc. injuries	Treatment	Follow-up
DiVincenti FC et al.	1969	Isolated vertebral body fractures in 2 patients	Electric shock	–	–	–
Rajam KH	1976	Non-specified fracture of vertebral bodies	Electric shock	–	–	–
Putti E et al.	1989	C5 fracture	Electric shock	–	–	–
van den Brink WA et al.	1995	L4 burst fracture	Low-voltage electric shock	Burn injury on the left hand	Plaster corset	3 months–consolidation without deformation
Sinha A et al.	2009	T4 compression fracture	Low-voltage electric shock	Right scapular fracture	Thoracolumbosacral orthosis	Persisting pain in the back

Several cases of bone fractures following LV trauma have been described in the literature. The most common fractures involved humerus and scapula [11] with the forearm being the third most common [12, 13].

Contrary, the incidence of bone injuries after a HV shock is much higher [5]. The mechanism usually involves contact with HV wires; however, iatrogenic VF following electric shock therapy [7] or cardioversion [14] have been described. Besides, tasers used by police can also cause muscle spasms resulting in VF [15].

## Conclusions

Albeit vertebral fractures caused by LV injury are extremely uncommon, clinicians should always consider this diagnosis in patients after an LV shock, especially when clinical symptoms are present. Quick and accurate diagnosis is the key element of full recovery. Therefore, we strongly recommend performing X-ray and CT scans in adult patients with symptoms; in children, we recommend performing X-ray and MRI scan.

## Data Availability

All data generated or analysed during this study are included in this published article (and its Additional files).

## References

[1] Schousboe JT (2016). Epidemiology of vertebral fractures. J Clin Densitom.

[2] den Ouden LP, Smits AJ, Stadhouder A, Feller R, Deunk J, Bloemers FW (2019). Epidemiology of spinal fractures in a level one trauma center in the netherlands: a 10 years review. Spine.

[3] Waterloo S, Ahmed LA, Center JR, Eisman JA, Morseth B, Nguyen ND, Nguyen T, Sogaard AJ, Emaus N (2012). Prevalence of vertebral fractures in women and men in the population-based Tromsø Study. BMC Musculoskelet Disord.

[4] El-Faramawy A, El-Menyar A, Al-Thani H, Zarour A, Maull K, Riebe J (2012). Presentation and outcome of traumatic spinal fractures. J Emerg Trauma Shock.

[5] Peyron PA, Cathala P, Vannucci C, Baccino E (2015). Wrist fracture in a 6-year-old girl after an accidental electric shock at low voltages. Int J Legal Med.

[6] Rana M, Banerjee R (2006). Scapular fracture after electric shock. Ann R Coll Surg Engl.

[7] Dewald PA, Margolis NM, Weiner H (1954). Vertebral fractures as a complication of electroconvulsive therapy. J Am Med Assoc.

[8] van den Brink WA, van Leeuwen O (1995). Lumbar burst fracture due to low voltage shock. A case report. Acta OrthopScand.

[9] Putti E, Tatò FB (1989). A case of fracture of the 5th cervical vertebra caused by electric shock. Chir Organi Mov.

[10] Vincenti FC, Moncrief JA, Pruitt BA (1969). Electrical injuries: a review of 65 cases. J Trauma.

[11] Stone N, Karamitopoulos M, Edelstein D, Hashem J, Tucci J (2014). Bilateral distal radius fractures in a 12-year-old boy after household electrical shock: case report and literature summary. Case Rep Med.

[12] Pappano D (2010). Radius fracture from an electrical injury involving an electric guitar. South Med J.

[13] Evans RJ, Little K (1991). Fracture due to shock from domestic electricity supply. Injury.

[14] Giacomoni P, Cremonini R, Cristoferi E, Guardigli C, Gulinelli E, Matarazzo V, Pancaldi S, Sgalaberna C, Valentini AM, Menghi B (1987). Vertebral fracture caused by electric cardioversion. G Ital Cardiol.

[15] Winslow JE, Bozeman WP, Fortner MC, Alson RL (2007). Thoracic compression fractures as a result of shock from a conducted energy weapon: a case report. Ann Emerg Med.

